# Sensitivity of three thyrotropin receptor antibody assays in thyroid-associated orbitopathy

**DOI:** 10.5937/jomb0-34718

**Published:** 2022-04-08

**Authors:** Marija Sarić-Matutinović, Tanja Diana, Biljana Nedeljković-Beleslin, Jasmina Ćirić, Miloš Žarković, Iva Perović-Blagojević, George J. Kahaly, Svetlana Ignjatović

**Affiliations:** 1 University of Belgrade, Faculty of Pharmacy, Belgrade; 2 Johannes Gutenberg University (JGU) Medical Center, Department of Medicine I, Molecular Thyroid Research Laboratory, Mainz, Germany; 3 University Clinical Center of Serbia, Clinic for Endocrinology, Diabetes and Metabolic Disorders, Belgrade; 4 University of Belgrade, Medical Faculty, Belgrade; 5 Clinical Hospital Center 'Dr Dragiša Mišović-Dedinje', Service for laboratory diagnostics, Belgrade; 6 University Clinical Center of Serbia, Center for Medical Biochemistry, Belgrade

**Keywords:** thyroid-associated orbitopathy, thyrotropin receptor antibodies, bioassay, bridge binding assay, ECLIA binding assay, orbitopatija udružena sa žtitnom žlezdom, antitela na receptor za tireostimulišući hormon, bioesej, "sendvič" imunohemijski test vezivanja, ECLIA test vezivanja

## Abstract

**Background:**

Thyrotropin receptor autoantibodies (TSH-RAb) are indispensable biomarkers in the laboratory assessment of thyroid-associated orbitopathy (TAO). Clinical sensitivity of three different assays for TSH-R-Ab determination was evaluated in patients with TAO.

**Methods:**

87 consecutive TAO patients were enrolled and their serum samples analyzed in parallel with three assays. An ECLIA competitive binding and a chemiluminescent bridge immunoassay were used to measure total and binding TSH-R-Ab concentration, while their functional activity was determined using a stimulatory TSH-R-Ab (TSAb) cellbased bioassay.

**Results:**

Compared to the two binding assays (ECLIA p<0.001, bridge p=0.003), the TSAb bioassay was more sensitive pertaining to the positive detection of TSH-R-Ab in TAO patients. No difference (p=0.057) was noted between the ECLIA and bridge assays regarding sensitivity rate. All patients with active and/or moderate-to-severe TAO tested positive in the TSAb bioassay (100% and 100%, respectively), while the positivity rates for bridge and ECLIA binding assays were 89.7% and 82.1% for active TAO, and 90.2% and 86.3% for severe TAO, respectively. Negative predictive values of the bioassay, bridge, and ECLIA assays were 100%, 75%, and 71%, respectively for active TAO, and 100%, 86%, and 71%, respectively for moderate-to-severe TAO. The superiority of the bioassay was most prominent in euthyroid (ET) TAO. Positivity rates of the TSAb bioassay, bridge and ECLIA binding assays were 89.6%, 75%, and 64.6%, respectively for inactive TAO; 86.1%, 69.4%, and 52.8%, respectively for mild TAO; 87.5%, 62.5%, and 12.5%, respectively for euthyroid TAO. The bridge assay correlated better with the ECLIA binding assay (r=0.893, p<0.001), compared to the bioassay (r=0.669, p<0.001).

**Conclusions:**

In patients with TAO of various activity and severity, the TSAb bioassay demonstrates a superior clinical performance compared to both ECLIA and bridge binding assays.

## Abstract (sr)

**Uvod:**

Autoantitela na receptore za tireostimulišući hormon (TSH-R-Ab) su nezamenljivi biomarkeri u laboratorijskoj proceni orbitopatije udru'ene sa štitnom žlezdom (TAO). U radu je procenjena klinička osetljivost tri različita testa za određivanje TSH-R-Ab kod pacijenata sa TAO.

**Metode:**

U studiju je ključeno 87 uzastopnih pacijenata sa TAO i njihovi uzorci seruma su analizirani paralelno sa tri testa. Za merenje ukupne i vezujuće koncentracije TSH-RAb korišćeni su ECLIA imunohemijski test kompetitivnog vezivanja i hemiluminiscentni imunohemijski "sendvič" test, dok je njihova funkcionalna aktivnost određena pomoću ćelijskog biološkog testa (bioeseja) za određivanje stimulatornih TSH-R-Ab (TSAb).

**Rezultati:**

U poređenju sa dva imunohemijska testa vezivanja (ECLIA P<0,001, "sendvič test" P=0,003), TSAb bioesej se pokazao najosetljivijim u pogledu pozitivne detekcije TSH-RAb kod TAO pacijenata. Nikakva razlika nije detektovana (P=0,057) između ECLIA i "sendvič" testa u pogledu stope osetljivosti. TSAb bioesej je bio pozitivan kod svih pacijenata sa aktivnom i/ili umerenom do teškom TAO (redom 100% i 100%), dok su stope pozitivnosti za sendvič i ECLIA imunohemijski test bile 89,7% i 82,1% za aktivnu TAO, i 90,2% i 86,3% za tešku TAO, redom. Negativne prediktivne vrednosti bioeseja, "sendvič" i ECLIA testova bile su redom 100%, 75% i 71%, za aktivnu TAO, odnosno 100%, 86% i 71%, redom za umerenu do tešku TAO. Superiornost biološke analize bila je najistaknutija kod eutiroidnog oblika (ET) TAO. Stope pozitivnosti TSAb bioeseja, "sendvič" i ECLIA testova vezivanja bile su redom 89,6%, 75% i 64,6%, za neaktivnu TAO; 86,1%, 69,4% i 52,8% za blagu TAO; 87,5%, 62,5% i 12,5% za eutiroidni TAO. "Sendvič" imunohemijski test je bio u boljoj korelaciji sa ECLIA testom kompetitivnog vezivanja (r=0,893, P<0,001), u poređenju sa bioesejom (r=0,669, P<0,001).

**Zaključak:**

Kod pacijenata sa TAO različite aktivnosti i težine TSAb bioesej pokazuje superiorne kliničke performanse u poređenju sa obe vrste imunohemijskih testova vezivanja (ECLIA i "sendvič test").

## Introduction

Thyrotropin receptor autoantibodies (TSH-R-Ab) are specific biomarkers of both Graves' disease (GD) and thyroid-associated orbitopathy (TAO) that define their pathogenetic background and clinical phenotype. They represent an indispensable diagnostic tool in the clinical assessment of GD and TAO [1] [2] [3] [4] [5] [6]. TSH-R-Ab express variable biological activity and are accordingly classified as stimulating (TSAb), blocking (TBAb), and neutral antibodies [7]. A timely and sensitive serological TSH-R-Ab testing is crucial for definitive diagnosis of GD and thyroid related orbitopathy [8]. Total TSH-R-Ab concentration, usually denoted as TSH-R binding inhibitory imunoglobulins (TBII), is quantified by competitive binding immunoassays traditionally widely used in routine laboratory diagnostics. It is a net sum of TSH-R-Ab of different variety and not representative of their biological activity. Chemiluminescent or radio labeled TSH or monoclonal antibody competes with TSH-R-Ab from patient's serum for binding to the same binding sites at TSH-R [9] [10]. To this day, two sorts of assays for detection of thyrotropin stimulatory antibodies have been developed and proposed as an alternative to the existing TBII testing: bioassays which measure the level of TSH-R-Ab functional activity (TSAb and TBAb) and recently developed bridge-immunoassay. The latter utilizes bridge immuno assay technology and reportedly detects TSH-R-Ab. This assay is intended for use on fully automated commercial platforms [11] [12] [13]. In contrast, the exclusivity of bioassay methodology is reflected in the ability to detect the biological function of thyrotropin autoantibodies. It uses genetically engineered CHO cells (Chinese hamster ovary cells), transfected with human TSH-R and cAMP-inducible luciferase reporter gene. Bioassays have been reported as highly specific and sensitive biomarkers of GD and TAO [14]. However, they are still not widely introduced into routine practice and require special laboratory conditions and well-trained staff.

Three major roles of a new diagnostic test have previously been defined: replacement (new test replaces an existing one), triage (new test is used before an existing one), and add-on (new test is used after an existing one). Thorough examination needs to preceed the implemantation of every medical test. Diagnostic accuracy assessment represents the major step when introducing new biomarkers and laboratory tests into routine clinical practice [15]. The intended clinical goal of the biomarker (early and accurate diagnosis, screening, prediction, prognosis, etc.) is a clear guidance for how the test will be used. Diagnostic accuracy is often balanced with the practical aspects involved as well. Apart from the superior diagnostic properties compared to the existing methodology other features must be also taken into consideration: invasiveness, cost, feasibility, availability, turnaround time, laboriousness, etc. [16].

The aim of this study was to perform a comparative analysis of the bioassay and bridge immunoassay for quantification of TSH-R-Ab, relative to the existing, commonly used competitive binding immunoassay. We intended to evaluate the diagnostic performance of the above mentioned laboratory tests, primarily in terms of diagnostic sensitivity, and accordingly to our findings propose the most appropriate replacement for the existing binding assay.

## Material and Methods

### Patients

This was an observational retrospective study. A total of 87 consecutive patients with clinically manifest TAO were recruited. All patients were regularly treated at the tertiary University Clinical Center of Serbia (Clinic for Endocrinology, Diabetes and Metabolic Diseases) according to the current guidelines and protocols [17] [18] [19]. Hormone analysis was carried out in all patients and the diagnosis was classified as Graves' disease (GD), Hashimoto's thyroiditis and euthyroid TAO. TAO was categorized according to its activity as active or inactive (seven points Clinical Activity Score (CAS), cut-off 3/7) and according to its severity as mild, moderate-to-severe, and sight-threatening TAO (current 2021 EU guidelines) [17] [20].

Upon admission, a detailed medical history and demographic data were taken. All participants signed an informed consent and Ethics Committee approval was obtained prior to the start of the study (17.06.2019/944/3). Research was conducted in accordance with the guidelines of clinical and laboratory practice, the Declaration of Helsinki and applicable institutional and national regulations.

Blood draw was performed in the morning, after 12 hours fasting period. After separation of serum, samples were appropriately aliquoted and stored at -80°C, until analysis. Samples were analyzed in parallel with all three different methods for TSH-R-Ab quantification.

### Conventional binding assay

Total TSH-R-Ab concentration was measured using a commercial automated binding immunoassay (ECLIA, Elecsys Anti-TSHR Immunoassay Roche Diagnostics, GmbH, Mannheim, Germany) on the Cobas e411 analyzer (Roche, Diagnostics, GmbH) according to the manufacturer's instructions (cut-off 1.75 IU/L). This assay employs a monoclonal M22 antibody with a high affinity for TSH-R-Ab but without the ability to distinguish their functionality.

### Cell-based bioassay

Serum TSAb and TBAb were measured using a commercial FDA-cleared bioassay (Thyretain, Quidel, San Die, CA, USA) and CE-marked bioassay, respectively, according to the manufacturer's instructions [21] [22]. Both tests utilize Chinese hamster ovary (CHO) cells that express chimeric TSH-R (Mc4) and cAMP-inducible luciferase reporter gene. When CHO cells are exposed to TSAb, cAMP-dependent production of luciferase occurs, which is quantified after addition of luciferin. In contrast, TBAb antibodies inhibit cAMP production and consequent light signal generation. Patients' samples and controls were added to CHO-Mc4 cells, previously seeded and grown in a 96-well plate. After incubation with CO 2 and cell lysis, a chemiluminescent signal was quantified as relative light units with a luminometer. TSAb level is expressed as percentage specimen-to-reference-ratio (cut-off 140 SRR%) and TBAb as percentage inhibition (cut-off 34% inhibition).

Serum TSAb and TBAb were measured in a blinded manner at the Molecular Thyroid Research Lab of the Johannes Gutenberg-University (JGU) Medical Center, Mainz, Germany where the samples were shipped on dry ice. The samples underwent only one thawing procedure and were analyzed with one reagent lot.

### Siemens bridge immunoassay

Commercial bridge immunoassay was used in this study (IMMULITE TSI 2000, Siemens Healthcare Diagnostics, UK), according to the manufacturer's instructions, on an IMMULITE 2000 analyzer. This is a fully automated, chemiluminescent immunoassay that employs the bridge assay format. It uses a pair of recombinant hTSH-R, where TSH-R-Ab from the sample binds through one arm to the immobilized capture hTSH-R and to the signal, alkaline phosphatase labeled hTSH-R, through the other arm, thus forming a bridge. The assay involves two cycles, the incubation with a capture receptor and the incubation with a signal receptor, with removal of the unwashed material in between. After addition of the chemiluminescent substrate to the reaction, the light signal is triggered and is measured by a luminometer. The signal is directly proportional to the TSH-R-Ab concentration in the sample [12] [13].

### Statistical analysis

Statistical analysis was performed using SPSS Software package version 20 (SPSS Inc., Chica, IL, USA). We assessed the normality of distribution with the Kolmorov-Smirnov and Shapiro-Wilk tests, depending on the sample size. We reported categorical variables as numbers or percentages, normally distributed continuous variables as mean ± standard deviation (SD), and non-normally distributed variables as median (interquartile range, IQR). Sensitivity rates among examined TSH-R-Ab assays were compared using the McNemara's test. Statistical significance was considered at a value of p<0.05.

## Results

Patients' demographic, serological, and clinical data are presented in Table 1. Out of 87 TAO patients, TSH-R-Ab positivity was detected in 82 (94.3%), 71 (81.6%), and 63 (72.4%) patients by TSAb bioassay, Siemens bridge binding assay, and Roche ECLIA binding assay, respectively (Table 2). Only one TAO patient showed both blocking and stimulating antibody activity. TSAb bioassay showed the highest sensitivity rate for detection of TAO in patients, meaning that it exerts the strongest ability to include diagnosis in patients with TAO. A significant discrepancy of the results was seen between both the TSAb bioassay and ECLIA binding assay (p<0.001), and between the TSAb bioassay and bridge binding assay (p=0.003), but not between the two binding assays (p=0.057) (Table 3). Overall concordance of the results was 85.1% for the bioassay and bridge assay, 83.9% for the bridge and ECLIA binding assay, and 78.2% for the bioassay and binding ECLIA assay. 12 TSAb bioassay positive samples were bridge assay negative and 1 TSAb bioassay negative sample was bridge assay borderline positive, what indicated the tendency of bridge assay towards a higher rate of »false« negative results. Interestingly, one GD patient with dual stimulating and blocking antibody activity tested negative in binding bridge assay, at the same time. All 19 discrepant TSAb bioassay/binding ECLIA assay results were TSAb positive and negative in the binding assay. None of TSAb negative patients were positive in the binding assay. 11 bridge binding assay positive samples were ECLIA binding assay negative, while 3 bridge assay negative samples tested positive in ECLIA binding assay.

**Table 1:** Patients’ demographic, clinical and serological, data TAO, thyroid-associated orbitopathy; GD, Graves’ disease; HT, Hashimoto’s thyroiditis; ET, euthyroid; TSH, thyroid stimulating hormone; FT4, free thyroxine; TSAb, TSH-R stimulating antibodies; The Thyretain TSAb functional bioassay cut-off is at 140 SRR% (specimen-to-reference ratio) and for Siemens bridge and Roche ECLIA binding assay cut-off is at 0.55 IU/L and 1.75 IU/L, respectively.

Parameter	All TAO patients
n	87
Age (years)	53±11
Gender (f/m)	52/25
TAO duration (years)	1.15 (0.67–3.00)
Diagnosis<br>GD+TAO (n)<br>HT+TAO (n)<br>ET+TAO (n)	67<br>12<br>8
Activity of TAO<br>active (n)<br>inactive (n)	39<br>48
Severity of TAO<br>moderate-to-severe (n)<br>mild (n)	51<br>36
Current smokers (n)	50
TSH (IU/L)	1.20 (0.10–3.26)
FT4 (pmol/L)	14.95 (13–19.37)
Thyretain TSAb functional bioassay	669 (298–764)
Siemens bridge binding assay	2.45 (0.71–10.52)
Roche ECLIA binding assay	3.87 (1.54–14.56)

**Table 2:** Positivity crosstabs for different TSH-R-Ab assays TAO, thyroid-associated orbitopathy; TSAb, TSH-R stimulating antibodies

	Thyretain TSAb functional bioassay		Siemens bridge binding assay			
	positive	negative	Total	positive	negative	Total
All TAO patients						
Roche ECLIA binding assay						
positive	63	0	63	60	3	63
negative	19	5	24	11	13	14
**Total**	**82**	**5**	**87**	**71**	**16**	**87**
TAO activity						
Active TAO						
Roche ECLIA binding assay						
positive	32	0	32	32	0	32
negative	7	0	7	3	4	7
**Total**	**39**	**0**	**39**	**35**	**4**	**39**
Inactive TAO						
Roche ECLIA binding assay						
positive	31	0	31	28	3	31
negative	12	5	17	8	9	17
**Total**	**43**	**5**	**48**	**36**	**12**	**48**
TAO severity						
Moderate to severe TAO						
Roche ECLIA binding assay						
positive	44	0	44	43	1	44
negative	7	0	7	3	4	7
**Total**	**51**	**0**	**51**	**46**	**5**	**51**
Mild TAO						
Roche ECLIA binding assay						
positive	19	0	19	17	2	19
negative	12	5	17	8	9	17
**Total**	**31**	**5**	**36**	**25**	**11**	**36**

**Table 3:** Distribution of the sensitivity rates among different TSH-R-Ab assays TAO, thyroid-associated orbitopathy; TSAb, TSH-R stimulating antibodies, SRR%, serum-to-reference ratio; TSAb (SRR%) low positive, TSAb level in TSAb bioassay ≤ 25^th^ percentile (140–298 SRR%), TSAb (SRR%) medium positive, TSAb level in TSAb bioassay 25^th^-75^th^ percentile(299–761 SRR%), TSAb (SRR%) high positive, TSAb level in TSAb bioassay >75^th^ percentile (>762 SRR%); Values are presented as a numberof subjects and percentage of positive results; ^a^p value for difference in the sensitivity rate between Thyretain TSAb functional bioassay and Siemens bridge binding assay in different patient groups, ^b^p value for difference in the sensitivity rate between Thyretain TSAb functional bioassay and Roche ECLIA binding assay, ^c^p value for difference in the sensitivity rate between Siemens bridge and Roche ECLIA binding assays

TAO patient group	Number of subjects(n)	Thyretain TSAb functionalbioassay	Siemens bridgebinding assay	Roche ECLIA binding assay	p value
		sensitivity (%)	sensitivity (%)	sensitivity (%)	
All TAO patients	87	94.3	81.6	72.4	0.003^a^, <0.001^b^, 0.057^c^
Active TAO patients	39	100	89.7	82.1	0.125^a^, 0.016^b^, 0.250^c^
Moderate-to-severe	51	100	90.2	86.3	0.063^a^, 0.016^b^, 0.625^c^
Inactive TAO patients	48	89.6	75	64.6	0.039^a^, <0.001^b^, 0.227^c^
Mild TAO patients	36	86.1	69.4	52.8	0.070^a^, <0.001^b^, 0.109^c^
< 1 year	42	100	90.5	81	0.125^a^, 0.008^b^, 0.219^c^
> 1 year	42	88.1	71.4	64.3	0.039^a^, 0.002^b^, 0.453^c^
TSAb (SRR%) low positive	17	100	47.1	41.2	0.004^a^, 0.002^b^, 1.000^c^
TSAb (SRR%) medium positive	44	100	93.2	81.8	0.250^a^, 0.008^b^, 0.125^c^
TSAb (SRR%) high positive	21	100	100	95.2	1.000^a^, 1.000^b^, 1.000^c^

All five TSAb bioassay negative patients were presented with mild, inactive form of TAO. Among 16 bridge binding assay negative results, 4 patients had active TAO and 5 patients showed moderate-tosevere form of disease. Out of 24 negative ECLIA binding assay results, 7 were detected in active TAO patients and 7 in moderate-to-severe TAO patients.

Next, we analyzed the clinical sensitivity of assays relative to the activity and the severity of TAO (Table 3). All active TAO patients were TSAb bioassay positive, indicating a 100% sensitivity and respectively, 47.6% and 100% positive (PPV) and negative predictive value (NPV) for detecting an active form of TAO. A statistically significant difference regarding sensitivity, i.e. discriminating ability to detect active TAO patients was observed between the TSAb bioassay and routine binding ECLIA assay, but not between two types of immunoassays. Siemens bridge and Roche ECLIA binding assays gave PPV and NPV of 49.3%, 50.8%, 75% and 70.8%, respectively. The difference in true positive fractions (dTPF) of the results obtained with TSAb bioassay and binding ECLIA assay was 17.9%, while relative true positive fraction (rTPF) ratio was 1.22. When tested for equality of the true positive fractions, we obtained a significant difference among these tests (p=0.016). dTPF between binding bridge assay and binding ECLIA assay was 7.7%, and rTPF was 1.1. P value of 0.250 suggested no significant difference in their true positive fractions.

When observing the inactive TAO patient group, the TSAb bioassay was significantly more sensitive than both immunoassays examined, whereas no significant difference was shown between two types of immunoassays (Table 3).

All moderate-to-severe TAO patients were TSAb bioassay positive, suggesting the recognition of moderate-to-severe cases of TAO with a 100% sensitivity and 62.6% PPV and 100% NPV. PPV and NPV for detecting moderate-to-severe TAO cases for bridge and ECLIA binding assays were 64.8% and 69.8%, and 86.3% and 70.8%, respectively. Relative to the bridge assay, TSAb bioassay was not significantly more sensitive in this patient group, but when compared to the routine binding ECLIA assay, bioassay was significantly more sensitive, unlike bridge assay. The difference in true positive fractions (dTPF) and relative true positive fraction (rTPF) were 13.72% and 1.16 for TSAb bioassay and ECLIA binding assay (p=0.016), and 3.9% and 1.05 for binding bridge and ECLIA assays, respectively (p=0.625).

In the mild TAO patient group no significant difference was observed in the sensitivity of bridge assay compared to neithter TSAb bioassay nor the ECLIA binding assay. However, TSAb bioassay was significantly more sensitive in relation to the commonly used ECLIA binding assay (Table 3).

Next, when we classified TSAb levels obtained by TSAb bioassay to low, medium, and high positive, we observed the largest discrepancy of results in the low-positive level TSAb group of patients (Table 3). In this group of patients, TSAb bioassay was the only one to express significant sensitivity rate with a median TSAb level above the defined cut-off value (TSAb bioassay 229 SRR% (182-273) vs. bridge binding assay 0.48 (0.14-1.45) IU/L vs. ECLIA binding assay 1.57 (0.85-2.68) IU/L).

Moreover, the difference in the clinical performance of the analyzed laboratory tests was particularly evident in ET TAO patients, where TSAb bioassay successfully detected 7 out of 8 (87.5%) ET TAO patients, bridge binding assay 5 out of 8 (62.5%), and ECLIA binding assay only 1 out of 8 (12.5%) ET TAO patients. Bridge assay sensitivity rate in this patient group did not significantly differ in relation to either bio assay (p=0.500) or ECLIA binding assay (p=0.125).

**Figure 1:**
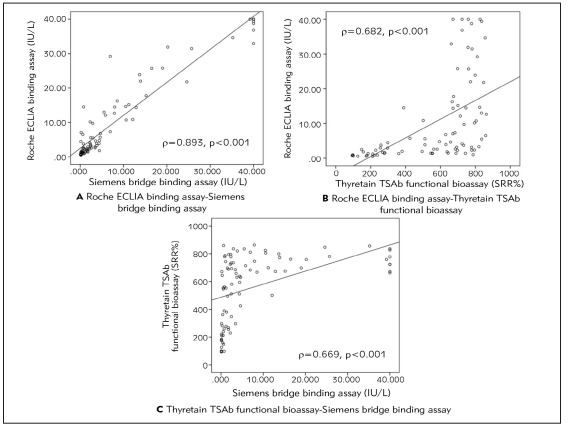
Various correlations between three different assays for TSH-R-Ab quantification in subjects with thyroid-associated orbitopathy

The bridge assay closely correlated with the ECLIA binding assay, significantly better than with the bioassay (r=0.893, p<0.001 vs. r=0.669, p<0.001). The bioassay correlated similarly with both binding immunoassays (r=0.669, r=0.682, p<0.001) (Figure 1).

Regarding the duration of TAO symptoms, superior TSAb bioassay sensitivity was the most prominent in TAO lasting over a year (Table 3). This is in line with the natural course of TAO, where the activity of TAO coincides with the more recent onset of disease [23].

Summary characteristics of all three analyzed TSH-R-Ab methods with special focus on the practical aspects are listed in Table 4.

**Table 4:** Comparative presentation of TSH-R-Ab test characteristics TSH-R-Ab, thyrotropin receptor autoantibodies; TAT, turnaround time

Method for TSH-R-Ab determination			
Test characteristics	Thyretain TSAb functional bioassay	Siemens bridge binding assay	Roche ECLIA binding assay
Accuracy	high	Moderate-to-high	Moderate-to-high
Invasiveness	Non-invasive	Non-invasive	Non-invasive
TAT	24h	Few hours	Few hours
Laboriousness	moderate	low	low
Sample type	serum	serum, plasma	serum, plasma
Sample preparation	No pretreatment	No pretreatment	No pretreatment
Interpretation of the results	Easy interpretation except incase of dual positivity	Easy interpretation	Easy interpretation
Cost	moderate	moderate	low
Feasibility	moderate	high	high
Automation	Semi-automatic test	Fully automated test	Fully automated test
Predictive potential	yes	-	No

## Discussion

The accuracy of a diagnostic method must be viewed from the perspective of the disease range and the context in which it is examined [24] [25]. Accordingly, the study design, as well as the choice of the appropriate statistical methods used, is usually defined by the intended purpose of the examined biomarker [16]. The present study focuses on the clinical value of a new replacement test relative to the standard, commonly used test for TSH-R-Ab detection. We compared the diagnostic features of the TSAb bioassay and bridge binding assay in relation to the standard competitive binding assay. We examined the variability of their performance both in all TAO patients and relative to the activity, severity, and duration of TAO. Replacement was chosen as the most suitable purpose for this assay comparison.

Superior diagnostic characteristics, primarily high sensitivity rate of the serological TSH-R-Ab methods are an imperative for accurate and timely differential diagnosis of TAO. Highly sensitive methods are necessary for the adequate recognition of a variety of TAO clinical phenotype. For clinicians, this feature is of utmost importance, since it defines further steps in patient management. 100% sensitivity and 100% NPV for detection of active and moderateto-severe TAO means that no such patient will remain undetected and that a negative test result will certainly exclude a progressive form of disease. These patients need to receive the appropriate therapy, and are more likely to develop sight-threatening TAO that requires an urgent treatment [17]. Moreover, a reliable and noninvasive serological test is a feasible first-line solution, especially if thorough clinical assessment in tertiary care units is not readily available. High sensitivity of TSH-R-Ab tests has particular clinical value in case of euthyroid TAO, a challenging clinical condition often confused with various other inflammatory disorders. Differential diagnosis of ET TAO is especially complicated and depends entirely on serological confirmation of TAO [26].

We have previously published our findings on the superior clinical performance of a TSAb cellbased bioassay versus the routine, binding assay. Bioassay showed 100% sensitivity for differentiating between active and inactive, as well as between mild and moderate-to-severe TAO patients, unlike binding assay that demonstrated significantly poorer discriminating ability [27]. Distinct difference between TSAb bioassay and binding ECLIA assay (p=0.031) was particularly prominent in ET TAO patient group. In this paper, we complement these previous findings in relation to the performance of the bridge assay. High analytical sensitivity of the bioassay technique was reported in a serial dilution analyses, where it demonstrated positivity at much higher serum dilution compared to the binding assays [28].

Our present findings demonstrate somewhat better diagnostic performance of the bridge binding assay compared to the traditionally used ECLIA binding test, but evidently poorer in comparison to the TSAb bioassay technique. Only in active TAO patients, the bridge assay performed similarly to the bioassay, although a small number of patients was involved. Interestingly, in neither of TAO patient groups, the bridge assay was significantly more sensitive than the ECLIA binding assay. In contrast, the functional bioassay showed markedly higher clinical sensitivity rate relative to the binding assay in all examined patient groups. The bioassay superior diagnostic sensitivity relative to both binding immunoassays was the most prominent in patients with milder clinical presentation of TAO (inactive TAO, low-positive TSAb). This suggest that the bioassay would be a better choice in management of atypical forms of TAO, without signs of inflammation and thyroid abnormalities [29]. In line with this, the variability of the clinical features was especially notable in ET TAO patient group, where only bioassay demonstrated a satisfactory positivity rate, i.e. the ability to detect virtually all ET TAO patients.

Dual stimulating and blocking antibody activity was observed in one GD patient. However, this patient tested negative in the bridge binding assay. Potential explanation lies in the variable affinity and concentration of TSAb and TBAb [30], possible mutual neutralization of the antibodies, as well as the specificity issues of the chimeric TSH-R construct used. TSAb and TBAb epitopes are not completely distinct entites, that is to say, they show a high level of overlapping. Naturally the TSAb and TBAb binding sites are located physically close to each other. TBAb binds to both Epitope B that is separated from TSAb binding site (Epitope A1), and Epitope A2 that is close to Epitope A1 [31] [32]. This suggests that the ability of an immunoassay to specifically measure stimulating antibody concentration and to accurately distinguish them from the blocking ones when present simultaniously in patient's sample, is a matter of debate and a problem for closer investigation [33]. In line with this, it was already reported that the bridge assay was not able to differentiate between TBAb and TSAb [34].

Routine binding assays are representative of the total TSH-R-Ab concentration, i.e. a net sum of stimulating, blocking, and even neutral antibodies. Bioassay measures the biological activity of TSH-R-Ab (through the level of cAMP) that is a direct cause of the clinical course of GD and TAO. In contrast, the receptor binding techniques measure the level of anti-body binding to the receptor which is highly dependent on the epitopes, antibody affinity, and concentration [33]. To this day, a commercial test for measurement of neutralizing TSH-R-Ab has not yet been developed [35]. According to manufacturer's claims, the bridge binding assay utilizes a recombinant human TSHR (MC4), allegedly specific for TSAb [13]. However, it was shown that MC4-expressing cell lines could be used for TBAb quantification as well [36] [37]. In fact the specificity of the bridge technology has never been proven and nonspecific TBAb detection was reported in multiple studies [34] [38] [39]. In an animal model of GD, developed by immunizations with extracellular domain of TSHR, the bridge assay could not distinguish TSHR functionality, as it yielded positivity in both TSAb and TBAb positive samples [40]. The bridge assay is therefore a purely binding assay, incapable of determining functional effect of TSH-R-Ab, meaning that bioassays provide wider information about the exact inflammatory status in TAO patients.

However, practical aspects must be kept in mind during the clinical validation of an assay, primarily the cost-benefit ratio of integrating new technologies into everyday practice [16] [41].

Long-term benefit of introducing functional biomarkers in routine clinical practice would be reflected in the reduced need for frequent hospitalization of patients and the use of expensive imaging diagnostic procedures [3] [42]. This could substantially alleviate the burden on the health care system. Introduction of highly sensitive serological markers would also minimize the use of radioactive iodine in the differential diagnosis of thyrotoxicosis, which is well associated to the progression of TAO.

In addition, unlike binding assays, functional biomarkers have demonstrated remarkable predictive value as indicators of relapse/remission of GD and the clinical phenotype of TAO [27] [43] [44]. This is another feature in favor of bioassays that would greatly facilitate patient monitoring and follow-up.

There are few limitations of our study: nonprospective design of the study and the impossibility of simultaneous analysis with all three laboratory tests for technical reasons. Nevertheless, this is one of very few studies to perform a comparative analysis of TSH-R-Ab assays and it was carried out at the referral, tertiary level clinic.

## Conclusions

In conclusion, we demonstrated superior clinical performance of the bioassay method compared to the traditionally used competitive binding ECLIA assay and the new bridge assay technique, primarily in terms of clinical sensitivity. Bridge assay performance was positioned somewhere in the middle and as such wouldn't be a suitable replacement for the commonly used binding method. In this way we strived to meet the clinicians' needs that are to maximize the sensitivity of the tests so as not to miss any TAO patient, especially those with mild and nonspecific presentation of the disease, as well as those who need to receive the appropriate treatment. According to these findings, as well as the clinical goals, we conclude that only the bioassay demonstrates sufficient diagnostic characteristics to replace the existing competitive binding assays where possible. Integration of bioassays into the current diagnostic algorithms of TAO could substantially improve patient management, monitoring, and prediction of clinical course of disease.

## Dodatak

### Acknowledgments

This research was fundedpartially by a grant No. 175036 of the Ministry ofEducation, Science and Technological Development,Republic of Serbia, and through Grant Agreementwith the University of Belgrade-Faculty of PharmacyNo: 451-03-9/2021-14/200161.

### Conflict of interest statement

All the authors declare that they have no conflictof interest in this work.
